# Measuring Cellular Immunity to Influenza: Methods of Detection, Applications and Challenges

**DOI:** 10.3390/vaccines3020293

**Published:** 2015-04-14

**Authors:** Lynda Coughlan, Teresa Lambe

**Affiliations:** The Jenner Institute, University of Oxford, ORCRB, Roosevelt Drive, Oxford OX1 7DQ, UK; E-Mail: lynda.coughlan@ndm.ox.ac.uk

**Keywords:** influenza, cellular immunity, T-cell, immunity

## Abstract

Influenza A virus is a respiratory pathogen which causes both seasonal epidemics and occasional pandemics; infection continues to be a significant cause of mortality worldwide. Current influenza vaccines principally stimulate humoral immune responses that are largely directed towards the variant surface antigens of influenza. Vaccination can result in an effective, albeit strain-specific antibody response and there is a need for vaccines that can provide superior, long-lasting immunity to influenza. Vaccination approaches targeting conserved viral antigens have the potential to provide broadly cross-reactive, heterosubtypic immunity to diverse influenza viruses. However, the field lacks consensus on the correlates of protection for cellular immunity in reducing severe influenza infection, transmission or disease outcome. Furthermore, unlike serological methods such as the standardized haemagglutination inhibition assay, there remains a large degree of variation in both the types of assays and method of reporting cellular outputs. T-cell directed immunity has long been known to play a role in ameliorating the severity and/or duration of influenza infection, but the precise phenotype, magnitude and longevity of the requisite protective response is unclear. In order to progress the development of universal influenza vaccines, it is critical to standardize assays across sites to facilitate direct comparisons between clinical trials.

## 1. Introduction

Influenza-associated morbidity disproportionately affects the younger child and older adult, with influenza-concomitant deaths occurring excessively in adults over the age of 65. There is an unmet need for sterilizing immunity toward Influenza A virus (IAV) and in parallel a, simultaneously growing, global healthcare burden influenced in part by an increasing and aging population [1,2]. Unfortunately, currently licensed influenza vaccines are limited in their ability to induce sterilizing immunity toward newly emergent influenza strains [3,4]; as evidenced by numerous influenza pandemics in 1918, 1957, 1968, 1977 and 2009. Neutralizing antibody responses to influenza viruses are largely directed towards the virus surface glycoproteins haemagglutinin (HA) and neuraminidase (NA). Reassortment combinations of the 18 HA or 11 NA proteins can result in a large number of influenza subtypes (e.g., H5N1 or H1N1), some of which can result in the emergence of mammalian-adapted influenza strains for which there is little or non-existing prior immunity in the human population. Influenza viruses evolve by the processes of antigenic shift, whereby specific HA or NA genes (along with others) are substituted for those derived from another subtype, which may be prevalent in an animal reservoir, or by antigenic drift, in which the highly error prone viral RNA polymerase can facilitate the accumulation of mutations within antigenic sites [5,6]. Natural exposure to influenza evokes a broad adaptive immune response which can confer protection toward variant influenza exposure [7,8,9]. However, the ongoing evolution of influenza viruses by antigenic shift and drift can allow evasion of these pre-existing, protective neutralizing antibody (NAb) responses. This remains a significant challenge for the development of broadly cross-protective NAb responses to influenza and limits pandemic preparedness.

## 2. T-Cell Immunity Cellular Immunity to Influenza Virus

Primary T-cell responses are induced when naive T-cells encounter antigen presented via MHC I or MHC II molecules (reviewed in [10]). During IAV infection, degradation of both folded viral proteins and defective ribosomal products facilitates loading of peptides (8–10 amino acids long) onto the HLA molecule [11,12]. Subsequent movement toward, and incorporation of the HLA loaded molecules into, the cell surface facilitates presentation of the viral antigens to circulating T-cells [13,14]. 

The initial recognition of antigen, in the presence of appropriate help, results in proliferation and differentiation of naive T-cells into effector and memory T-cells, a process which is critical in shaping the type and magnitude of the ensuing antigen-specific immune response and subsequent memory response [15,16,17]. The principal role of IAV-directed cytotoxic T-cells (CTLs) is the recognition, and subsequent elimination, of IAV-infected cells, thus preventing the production of progeny virus [18]. As such, CTLs can curtail symptoms associated with viral replication and reduce the duration of subsequent infections but cannot inhibit initial IAV infection. 

IAV-specific CD8^+^ CTL cells predominantly recognize internal proteins [19] and in particular the abundantly expressed M1 and NP [20]; there is a high degree of conservation in both NP and M1 for different influenza isolates, suggesting stringent sequence constraints within these internal antigens [16]. IAV-specific CTLs are thought to promote viral elimination and host recovery through cytokine-directed killing of virus-infected cells, with some evidence for Fas-FasL mediated killing [16,21]. Mechanistically, these cytokines can include IL-2, TNF-α, IFN-γ and cytolytic enzymes perforin and granzyme [16]. The differentiation potential of effector T-cells is partially determined by initial antigen encounter [22] and broadly speaking CD8^+^ T cells can be classified as Tc1, Tc2 or Tc17 depending on cytokine production profile. Tc1 and Tc2 have direct cytolytic capacity which is not the case for the Tc17 subset (reviewed in [23]). However, it has been demonstrated that in addition to the classical IFN-γ producing Tc1 cells, IL-4 producing Tc2 and IL-17 producing Tc17 cells are able to provide protection to influenza virus infection [24,25]. In addition, CD4^+^ T-cells have been demonstrated to directly kill virally infected cells and have been implicated in the clearance of IAV [26]. CD4^+^ T-cells can also provide help to both CD8^+^T-cells and, most importantly, the humoral arm of the immune system—critically important in preventing IAV infection [27,28]. 

After IAV clearance, variant memory T-cell populations are established which can provide protection against secondary infection [29]. Memory T-cells can be broadly classified into four groups, central memory T cells (T_CM_ cells), effector memory T cells (T_EM_ cells), tissue-resident memory T cells (T_RM_ cells) and stem memory T cells (T_SCM_) [30]. Circulating memory T-cells are generally either effector memory (T_EM_) or central memory (T_CM_) populations, which may be distinguished by location. In addition, and perhaps playing a more crucial role in tempering infectious disease are non-circulating tissue-specific memory CD8^+^ T cells (T_RM_) [30,31]. It has been demonstrated that lung resident memory T cells are a critical component of heterosubtypic immunity toward influenza virus and a decline in CD8^+^ T_RM_ correlates with a loss of protection from disease [32]. While T_RM_ are abundant in human lung, and are responsive toward influenza virus, their exact role during real-life human infection remains to be determined [33]. 

## 3. Cellular Immunity in Human Infection

A protective role for CD4^+^ and CD8^+^ T-cells during IAV has been demonstrated in mice [34] and has been confirmed in larger animal models including, the pig, ferret and non-human primates [15,16,35]. Consistent with observations of T-cell mediated protection in pre-clinical models of IAV, historical data from the Cleveland Family Study suggest that only 5.6% of the adults who had had symptomatic IAV in earlier years developed influenza during the 1957 pandemic, suggesting a protective effect of previously established influenza-specific memory T cells [36]. However, 55.2% of children who had had symptomatic IAV disease contracted it again, suggesting an impact of accumulated heterosubtypic immunity during pandemic IAV exposure [36]. Indeed, recent exposure to seasonal viruses conferred a degree of T-cell protection toward the 2009 pandemic IAV [37,38,39,40,41]. Work carried out in the early 1980’s by McMichael *et al.*, established a role for T-cells in mitigating influenza-associated illness in human challenge studies [7]. Volunteers with low HI titres toward influenza A/Munich/1/79 virus were exposed to live unattenuated virus by nasal administration. All subjects with measurable T-cell responses cleared virus effectively and were conferred a degree of protection from influenza challenge [7]. 

Some 30 years later, technological advances have facilitated a more thorough delineation of the T-cell populations associated with lessening influenza-associated morbidity [42,43]. During the 2009 pandemic waves in the UK, it was demonstrated that higher frequencies of pre-existing IFN-γ^+^ T-cells to conserved CD8^+^ epitopes were found in individuals who developed less severe illness [43]. In addition, more recent influenza challenge studies have demonstrated a correlation with IAV-specific CD4^+^ T-cells and lower virus shedding with less severe illness in humans who were experimentally infected with non-virulent IAV strains. While the experimental challenge model highlights the importance of CD4^+^ T-cells during IAV in humans a correlation was not seen with this T-cell population during the 2009 IAV pandemic. This may reflect inherent differences in prior exposure to influenza, dose of infection and/or may also reflect differences in T-cell assay design [42,43]. Nevertheless, there is collective evidence demonstrating that both IAV-specific CD4^+^ and CD8^+^ T-cells afford cross protective immunity toward various subtypes of IAV. Indeed, *ex vivo* cellular immune responses to IAV are correlated with protection in the older adult [44] and preliminary studies of young children confirmed that the IFN-γ ELISPOT assay was a more sensitive measure of influenza memory than serum antibody titers [45] . 

## 4. Current Influenza Vaccines

Currently licensed influenza vaccines include the trivalent or quadrivalent inactivated vaccines (TIV/QIV) which include two Influenza A subtypes and one or two Influenza B subtypes. These vaccines largely aim to stimulate humoral immune responses to HA and NA. When TIV is well matched to circulating influenza strains, vaccine efficacy can reach 75% [46,47]. However, mismatches to drift variants can limit the protective effects of TIV, particularly in vulnerable groups such as the elderly [48]. Furthermore, the efficacy of TIV and QIV in children is ~59% [49,50]. Clearly, there is a need for alternative approaches to influenza vaccination, particularly within at-risk groups. Unlike influenza HA and NA, which are subject to intense selective pressure to mutate and evolve, internal influenza antigens such as nucleoprotein (NP) and matrix protein-1 (M1) are more highly conserved among multiple influenza subtypes [51]. In addition, these antigens are expressed abundantly in influenza infected cells [52] and are processed and presented to T-cells via the MHC pathway, making them good vaccine targets for stimulating cellular immune responses. The live attenuated influenza vaccine (LAIV), capable of limited replication in the upper respiratory track and thus triggering cell mediated immunity (CMI), has been licensed and has demonstrated good efficacy (64%–93%) in children aged between 2 and 7 years, although efficacy in adults aged between 18–49 ranged from 8%–48% [53]. Subsequently, the development of novel vaccines that boost naturally acquired T-cell immunity would yield enormous benefits and is the central tenet of many “universal” influenza vaccines under clinical development.

## 5. T-Cell Vaccination Approaches

Aside from the currently licensed LAIV influenza vaccine, several other types of vaccines which are capable of inducing CMI responses are under clinical investigation. These include viral vectored vaccines (replication competent or non-replicating) or plasmid DNA-based vaccines, all of which can be used in homologous or heterologous prime-boost regimens. Due to their demonstrated safety and immunogenicity in clinical trials [10,54], adenoviral and poxviral vectors are widely used for vaccine development for a broad range of disease targets [55,56,57,58], including influenza [59,60,61,62,63,64]. Regulatory authorities have acknowledged the importance of developing novel vaccination approaches for influenza [65,66], and strategies which stimulate cellular immunity could be particularly attractive from a public health and economic perspective, as they may help to limit disease severity, influenza-related hospitalizations and worker absenteeism [66,67]. However, although many of the assays employed in measuring cellular immunity are well established, the field lacks uniform and standardized protocols, which complicates the interpretation of data obtained from different laboratories. The future development and widespread licensure of T-cell vaccines will require the implementation of standardized assays which provide clear correlates of protection or measures of vaccine efficacy in clinical studies.

## 6. Techniques Used to Quantify Cellular Immunity

Traditional and widely-used assays measure T-cell function by (1) detection of cytokine responses (e.g., ELISA, ELISPOT); (2) phenotyping T-cells (e.g., flow cytometry); (3) assessing T-cell proliferation in response to antigen (e.g., ^3^H-thymidine incorporation or carboxyfluorescein succinimidyl ester CFSE); (4) determining antigen-specific cytotoxicity (e.g., chromium release assay) as well as (5) novel systems biology approaches which include differential gene or microRNA expression. Technological advances in recent years have resulted in the generation of numerous novel T-cell assays, such as the improved FLUOROSPOT ELISPOT assay which can detect multiple cytokines in the same well, or new approaches such as cytometry by time-of-flight mass spectrophotometry (CyTOF) which has the capacity to measure >50 parameters simultaneously. However, each method has its advantages and disadvantages and there remains a high degree of inter-laboratory variability in technical approaches, interpretation and identification of end-points that correlate with protection severe influenza illness.

### 6.1. Cytokine Based Assays

#### 6.1.1. ELISA and Multiplex Cytokine Assays

The measurement and analysis of cytokines released by T-cells in response to specific pathogens and/or vaccine antigens can provide invaluable information about the host immune response in disease [68]. As a result, a large number of T-cell assays are designed to monitor cytokine responses. Measurement of the frequency of IFN-γ producing T-cells is most widely used [69] as a result of the role of IFN-γ in the clearance of numerous pathogens, although TNF-α and IL-2 are also frequently measured [70]. Approaches for measuring cytokines include the classical enzyme-linked immune-sorbant assay (ELISA) which measures cytokines associated with defined T-cell subsets. One disadvantage of this approach is that typically only one protein can be measured at a time and relatively large sample volumes are required [71]. 

An improved approach is the cytometric bead array (CTA), a multiplex immunoassay which can allow the simultaneous quantification of multiple cytokine/chemokines. The advantages of this technique are that as little as 25–50 µL sample volumes are required and the assay is sensitive, capable of detecting soluble analytes at concentrations of <0.3 pg/mL. Analysis can be performed by flow cytometry using dedicated software or using specialised platforms such as the Luminex xMAP system, which can analyse up to 500 analytes per sample, including cytokines/chemokines, intracellular proteins, growth factors and phosphorylated cell signalling molecules. The CTA is based on the use of capture beads, each with a discrete fluorescence intensity which is customised by coating beads with a specific capture antibody (e.g., anti-IFN-γ). The sample is incubated with the beads and a mixture of fluorescently conjugated detection antibodies are added, before the sample is washed and data acquired using a flow cytometer. As with the ELISA assay, results are compared to a standard curve of known protein concentration for each analyte.

#### 6.1.2. ELISPOT Assays

**Figure 1:**
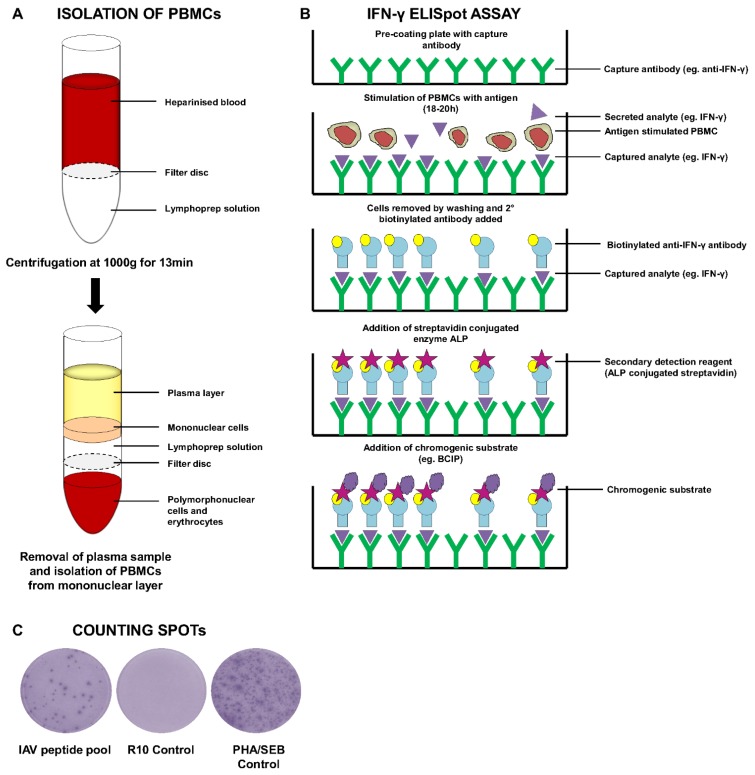
Schematic overview of PBMC isolation for IFN-γ ELISpot Assay. (**A**) Heparinised blood is added to lymphoprep-containing leucosep tubes and samples centrifuged for 13 min at 1000 ×*g* without brake. A plasma sample is taken before mononuclear/PBMCs are collected. Cells are washed and counted before being used for an ELISPOT assay; (**B**) Schematic diagram outlining the methodology behind an IFN-γ ELISpot assay; (**C**) Example of spot forming units following development of an ELISpot assay, showing antigen-specific IAV responses, negative control medium alone (R10) and positive control (PHA/SEB).

Alternatively, the ELISPOT assay (Figure 1A–C) is an extremely sensitive and accurate method for detection of antigen-specific T-cells (or B-cells), allowing for identification of a single cell secreting a particular cytokine (e.g., IFN-γ). The technique was first developed by Czerkinsky and colleagues in the 1980s [72] and has subsequently been accepted as one of the most validated assays for human clinical trials [73,74]. The method involves the isolation of PBMCs and addition of a set number of cells to a capture antibody-coated plate (e.g., anti-IFN-γ). Cells are then stimulated with a pre-determined concentration of specific antigen (e.g., peptide, virus or whole protein antigen). In the presence of the stimulus, antigen-specific T-cells will secrete cytokine (e.g., IFN-γ) which can be captured by the antibody used to coat the plate. Following an assay-dependent period of stimulation (usually 18–20 h), the cells are removed by washing and the bound cytokine typically detected using a secondary detection reagent conjugated to an enzymatic label (e.g., alkaline phosphatase—ALP). This enzyme catalyzes the colorimetric spot formation when in the presence of a chromogenic substrate (e.g., 5-bromo-4-chloro-3'-indolyphosphate p-toluidine salt—BCIP). Other enzymes which can be used for development of ELISPOTs include horseradish peroxidase, followed by addition of the chromogen 3,3',5,5'-tetramethylbenzidine (TMB). The choice of controls is also of critical importance in the ELISPOT assay. Most immunologists choose to stimulate with the polyclonal mitogen phytohaemagglutinin (PHA) as a positive control for the assay. However, Cytomegalovirus/Epstein Barr virus (CMV/EBV) peptide pools which consist of epitopes presented by broad range of HLA alleles are also included for quality control and standardisation purposes [75]. The use of whole antigen or peptide pools spanning large antigens ensures that the ELISPOT assay is not limited by HLA restriction to the same extent as ICS-based tetramer assays (see below). Furthermore, the use of the ELISPOT assay is attractive because it allows enumeration of the number of antigen-specific cells through calculation of the number of spot forming units (SFU). Additionally, the assay can be used on fresh or frozen PBMCs [76].

In recent years, there have been several adaptations and improvements to the standard ELISPOT assay, namely the development of a multiplex ELISpot assay which permits the simultaneous detection of multiple cytokines [77,78]. The Fluorospot assay can be used to detect single T-cells which secrete multiple cytokines and has been shown to offer improved characterisation and discrimination between double-cytokine positive cells when compared to an enzymatic multiplex ELISPOT assay [78]. Importantly, this new approach has been shown to retain the sensitivity of a conventional ELISPOT assay [78]. The methodology is largely similar to that described above for the standard IFN-γ ELISpot assay but differs in the use of multiple cytokine-specific capture antibodies when coating the plate. In addition, development of the assay included the use of secondary detection reagents coupled to different fluorophores such as phycoerythrin (PE) and Alexa Fluor 488. Spot forming units are then counted using a plate reader equipped with the capacity to read fluorescence.

An additional adaptation of the ELISPOT assay is the 10–14 day cultured ELISPOT assay, which has been shown to be an important tool in the detection of memory T-cell responses [79] and elucidating correlates of protection [80,81] or improved outcome in many infections [82,83]. The assay is similar to the standard ELISPOT assay, albeit with the addition of recombinant human IL-2 to the culture medium on days 3 and 7 post-culture [69]. However, there are many variations on this protocol which include duration of culture or the concentration and frequency of IL-2 supplementation. It is important to note, that there are differences between the responses obtained from the standard and cultured ELISPOT assay, which is to be expected considering that cytokine production from different T-cell populations are being measured by both assays. More detailed characterisation of these differences may help to improve understanding of the relationship between effector and memory T-cell responses to defined antigens [79].

### 6.2. Flow Cytometry/Cell Phenotyping Assays

Flow cytometric approaches are ideal for characterising the functional heterogenicity of T-cell responses and can be used to simultaneously quantify magnitude, phenotype and to indicate T-cell function.

#### 6.2.1. Tetramer Assay

**Figure 2:**
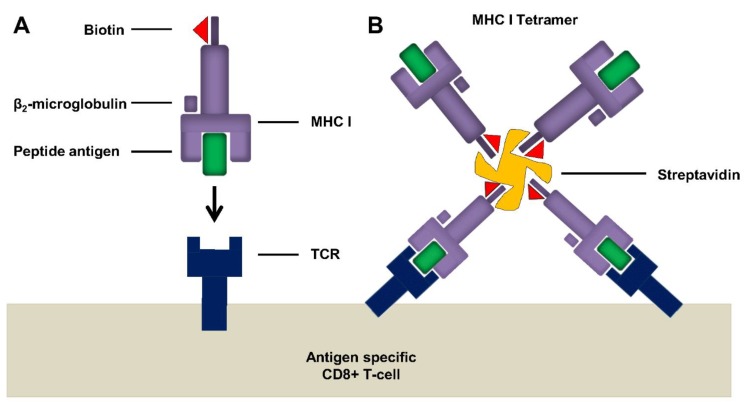
Tetramers increase avidity for epitope specific TCR interactions. (**A**) Schematic showing the recognition of peptide loaded biotinylated MHC I by Ag-specific TCR. Individual molecules have weak binding and cannot be stained; (**B**) Tetramerization of MHC I subunits is achieved by addition of fluorescently labelled streptavidin which interacts with biotin. Tetramerization increases avidity, TCR specificity and allows detection of Ag-specific T-cells by flow cytometry. Adapted from Klenerman and colleagues [84].

Tetramers (pentamers, hexamers or dextramers are also available) are synthetic MHC complexes made up of four or more identical versions of HLA molecules held together by non-covalent interactions (Figure 2). Each subunit is biotinylated, and tetramerization is facilitated by conjugation to a fluorescently-labelled streptavidin molecule [84]. These homo-tetrameric complexes are subsequently loaded with an antigen-specific peptide, allowing them to specifically identify and label CD8^+^ T-cells which express T-cell receptors (TCRs) specific for that peptide-MHC complex. The tetrameric nature of these complexes increases the affinity of the interaction between MHC I-TCR due to their ability to bind up to three separate TCRs simultaneously. Steric constraints can affect the ability to engage all four peptide complexes [85]. Specialised tubes, such as BD TruCOUNT^TM^ tubes, can facilitate the accurate enumeration of absolute leucocyte counts and following analysis using flow cytometry, antigen-specific responses can be quantified as a percentage of total CD8^+^ T-cells. Although, it is important to note that not all tetramer-positive cells represent functional antigen-specific T-cells. Tetramer assays are specific and sensitive and can be combined with other functional assays thus facilitating the enrichment or isolation of rare antigen-specific T-cell populations. Indeed, cloning of low frequency antigen-specific T-cells has been demonstrated in the past using HLA-A2.1-restricted CTL clones recognising the influenza matrix protein peptide 58–66 [86,87]. In addition, tetramer assays have been shown to have minimal intra-assay variation, better precision and linearity than ICS or ELISPOTs performed using frozen PBMCs from the same donors [88].

The disadvantages of using tetramers are that they are epitope-specific, thus, depending on the disease of interest, may require epitope mapping and knowledge of the HLA status of the donor subject. However, a great deal of information on T-cell recognition of influenza epitopes is available, thus facilitating the use of tetramer-based technologies [89,90,91]. Historically, tetramer labelling has proven more difficult to develop for CD4^+^T-cells for a number or reasons, namely the diversity of MHC allelic variants, the poor characterisation of epitopes, issues with detection of low-frequency antigen-specific CD4^+^T cells and the biodistribution of CD4^+^cells of interest [85,92]. 

#### 6.2.2. ICS Intracellular Cytokine Staining

Another method which permits identification of antigen-specific, low frequency, cytokine secreting cells on a single cell basis is intracellular cytokine staining (ICS), a flow cytometry based approach which, unlike ELISA or CTA, measures cytokines from an entire cell population and may include individual cell phenotyping [93,94]. PBMCs are stimulated with a specific antigen in the presence of a Golgi inhibitor (e.g., Brefeldin A or monensin), which acts as protein transport inhibitor preventing the secretion of cytokines, allowing cytokines to be retained intracellularly for subsequent detection by intracellular staining. Cells are fixed before being permeabilized, followed by incubation with fluorescently conjugated anti-cytokine antibodies which can now access the trapped cytokines. Additional antibody panels can be used in combination to identify specific cell subsets such as Th1, Th2, Th17 CD4^+^ T-cells, or can be classified as Tc1, Tc2 or Tc17 CD8^+^ T-cells and populations of variant memory T cells (e.g., central memory, effector memory, tissue-resident memory and stem memory T cells). This list of T-cell subsets is not exhaustive and other populations do exist (reviewed in detail in [95,96,97]), but are beyond the scope of this review.

The functional polarization of T-cell populations is driven by a combination of the effects of local cytokine milieu, costimulatory molecules, host genetic factors and to some extent, the strength of the interaction between the antigen and TCR [98]. T-cell subsets can typically be distinguished on the basis of their cytokine secretion profiles, a factor which has been exploited commercially, with antibody cocktail kits now specifically designed to enable their easy distinction and simultaneous phenotyping. For example, it is known that CD4^+^ Th1 T-cells, whose proliferation is triggered by IL-2 and IL-12, largely produce IFN-γ as an effector cytokine [96]. The secretion of IFN-γ, along with other effector molecules, stimulates CD8^+^ T-cells and promotes the phagocytic activity of macrophages [99], which are important drivers of cell-mediated immune responses and can lead to the successful elimination of intracellular bacteria, viruses and protozoa. The development of CD4^+^ Th2 responses is assisted by IL-4 and the signature effector cytokines produced include IL-4, IL-5, IL-9, IL-10 and IL-13. The downstream effector cells of Th2 T-cells include eosinophils, basophils, mast cells, B cells, and IL-4/IL-5 responsive CD4^+^ T cells [97]. Th2 T-cells play a key role in immunity to extracellular pathogens, particularly helminths. Th17 CD4^+^ T-cells, which are characterised by the production of IL-17A [97], and play a key role in protective immunity toward extracellular bacteria and fungi [96].

In a similar manner to CD4^+^ T-cells, CD8^+^ T-cells can also differentiate into unique and identifiable subsets, Tc1, Tc2 and Tc17 [95]. As with Th1 CD4^+^ T-cells, Tc1 CD8^+^ T-cells also largely produce high levels of IFN-γ. The cytokine profile associated with CD8^+^ Tc2 cells almost completely overlaps with those produced by Th2 cells, as they produce IL-5, and IL-13, although, limited levels of IL-4 [95]. As their name suggests, CD8^+^ T_C_17 cells are characterized by expression of the cytokine IL-17.

Multifunctional T-cells, which are capable of simultaneously secreting various combinations of cytokines, and their role in vaccine efficacy have been the subject of much interest in recent years. It has been suggested that multifunctional T-cells capable of simultaneously secreting combinations of IFN-γ, TNF-α and IL-2, play an important role in the control of Hepatitis C virus (HCV) [100] and Human Immunodeficiency Virus (HIV) [101]. The latter study demonstrated that triple producer CD4^+^ T cells (IFN-γ, IL-2 and TNF-α) showed an inverse correlation with viral load. Importantly, triple producing multifunctional T-cells have also been implicated in vaccine-mediated protection against *Leishmania major* in mice [102]. In humans, multifunctional T-cells have been shown to contribute to the efficacy of smallpox vaccines [103] and have also been proposed to play an important role in vaccination against *Mycobacterium tuberculosis* (TB) using BCG and viral vectors [104], although the relevance of polyfunctional CD4^+^ T-cells in serving as a correlate of protective immunity for TB has recently been challenged [105]. The continued delineation of phenotype and function of the many T-cell subsets, will undoubtedly enable researchers to identify immune correlates of protection in disease and following vaccination. It is becoming increasingly apparent that the unidirectional non-reversible differentiation of T-cell lineages is questionable and that functional and phenotypic plasticity should be appreciated when defining T-cell populations.

#### 6.2.3. Whole Blood Assay

Analysis of cytokine responses by flow cytometry can also be assessed following antigen stimulation in whole blood samples [93,94,106,107]. This approach offers a number of advantages over traditional methods of cell isolation, including the capacity to measure immune responses in a more physiologically relevant system which contains autologous serum and other cellular components. It can also be used to quantitate the frequency of CD4^+^/CD8^+^ T-cells in small volumes of whole blood, a factor which is particularly important in studies involving children or in field studies as the technique does not require equipment or reagents to separate peripheral blood mononuclear cells (PBMCs). Importantly, the whole blood assay has been shown to be sensitive and specific for detection of mycobacteria-specific immunity [107]. This assay is also capable of measuring cytokine responses to Epstein-Barr virus (EBV) [108], Human Immunodeficiency Virus (HIV) [109] and has also been shown to offer improved sensitivity in the detection of the endotoxic activities of lipopolysaccharide (LPS) derivatives [110]. Importantly, the whole blood assay was successfully used to narrow down individual peptide HIV-specific responses by using 396 overlapping synthetic peptides spanning the entire HIV genome arranged in a pool and matrix design, from only 16 mL heparinized blood [109]. This approach could also be used in studies which aim to characterise cellular immunity to influenza in the future.

**Figure 3:**
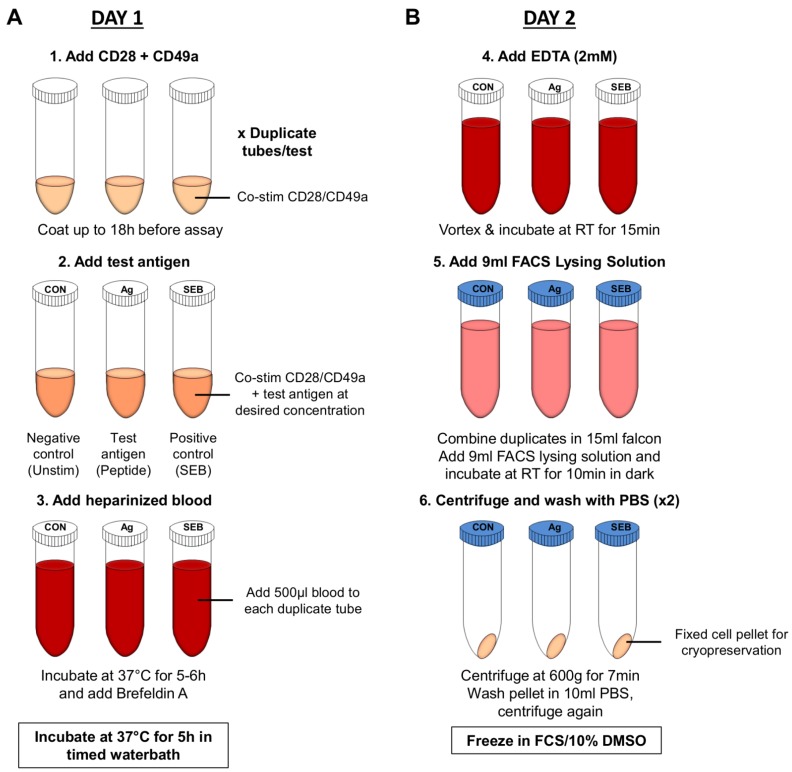
Schematic overview of Whole Blood Assay (**A**) Day 1: whole blood samples are stimulated with antigen, protein export inhibited and samples incubated overnight; (**B**) Samples are prepared for cryopreservation. Red cells are lysed and stimulated cells frozen down for future analysis by flow cytometry.

The specific details of individual protocols such as choice of stimulating antigen, concentration of antigen and controls to measure responses to different pathogens require optimization; however the general procedure is relatively similar. In brief, published protocols involve pre-coating polyprolypene tubes with CD28 and CD49a co-stimulatory antibodies which enhance the specific immune response [111], by increasing the observed frequency of cytokine responding CD4^+^T-cells in response to antigen [111]. Heparinized whole blood and antigen is added at an assay-dependent concentration and samples incubated for 5–6 h at 37 °C after which Brefeldin A and/or monensin is added to help retain cytokines intracellularly (e.g., IFN-γ). Samples are then incubated at 37 °C for a further 5 h after which EDTA is added to detach T-cells from the polyprolypene wall, erythrocytes are lysed and white cells fixed and cryopreserved in foetal calf serum (FCS) containing 10% DMSO (Figure 3). Following cryopreservation, phenotyping of cells by flow cytometry using tetramer staining or ICS can be performed off site where resources and specialised equipment are more readily available. 

### 6.3. Proliferation Assays

The proliferation of PBMCs following stimulation in cell culture with an antigen can be used to measure the induction of CMI. The incorporation of nucleosides into DNA can be measured and detected using three methods, (i) radioactivity; (ii) antibodies or (iii) click-chemistry. One example of such a proliferation assay is based on the incorporation of ^3^H-thymidine, a radioactive nucleoside into chromosomal DNA during mitosis. A scintillator counter is then used to measure the radiation output from the sample. However, this technique is slow, laborious and involves work with potentially hazardous radioactive materials. Therefore, similar assays have been developed using antibody-mediated detection of 5-bromo-2'-deoxyuridine (BrdU) as an alternative to the use of radioactive isotopes. Novel methods such as the click chemistry–based Click-iT^®^ 5-ethynyl-2'-deoxyuridine (EdU) assay [112,113], a modified nucleoside which is incorporated during DNA synthesis, have been developed more recently. Incorporation of EdU into proliferating cells is detected via visualization of a fluorescent azide following a copper(I) mediated chemical reaction. The advantages of this technique are that it is rapid, highly sensitive and in addition to facilitating analysis by flow cytometry, permits detection of DNA synthesis in whole tissue sections or explants and does not require sample fixation of DNA denaturation. Detection of proliferation using this method is also compatible with other fluorophores used routinely in flow cytometry, thus enabling simultaneous detection of other cellular parameters. However, some applications can benefit from the incorporation of two different analogs at different time points known as dual pulse labeling. Advantageously, the BrdU labeling technique can be combined with click chemistry detection for a simplified method of dual pulse labeling [114]. Another alternative method which is suitable for detection by flow cytometry and also eliminates the need to use radioactivity is the use of carboxyfluorescein succinimidyl ester (CFSE) to fluorescently stain cells. Following antigen stimulation, T-cell proliferation is initiated, resulting in the fluorescent CFSE intensity being halved with each cell division. These divisions or halving in fluorescence intensitycan be tracked by flow cytometry for up to 8 daughter cell divisions. Unlike radioactivity based proliferation assays, this approach can be combined with cell phenotyping. 

### 6.4. Cell Killing or Cytotoxicity Assays

Functional assays to measure cytotoxic T-cell killing capacity are based on the measurement of markers of cellular apoptosis or necrosis in target cells. The chromium release assay (^51^Cr), developed in the 1960s, has traditionally been used to measure antigen-specific CTL activity [115]. Target cells, loaded with an antigen of interest (e.g., MHC I-restricted peptide), are pre-labelled with 51-chromium which is released following cytolysis of the cells mediated by effector cells. ^51^Cr released into the supernatant is measured using a gamma counter. Different ratios of effector cell to target cells (known as E/T ratio) are routinely used and need to be optimised depending on the cell type used. Although this assay is highly reproducible and easy to perform, it has a low level of sensitivity, does not provide information about individual cell phenotype and there are also issues with the disposal of radioactive materials [116]. As with proliferation assays, numerous alternative assays exist which do not require the use of radioactivity. These include the measurement of enzymes such as lactate dehydrogenase (LDH) [117] or endogenous alkaline phosphatase [118] which are released into the cell supernatant following cytolysis by antigen-specific effector cells. Alternatively, fluorometric methods such as the calcein-acetoxymethyl (Calcein-AM)-release assay can be used as an alternative to the ^51^Cr assay [119]. In this assay, target cells are pre-labelled with the cytoplasmic fluorescent label, co-incubated with effector cells and fluorescence of dead/dying cells is quenched with the FluoroQuench reagent, before viability is read using a quantitative fluorescent scanner. Similar results to the latter assay can be achieved by transfecting/transducing cells with antigen conjugated to fluorescent proteins including green fluorescent protein (GFP), either using viral vectors or plasmid DNA [120].

Similar to proliferation assays, flow cytometry offers a number of advantages in its capacity to simultaneously measure CTL function as well as cell phenotype. A number of approaches can be used including the detection of apoptosis in target cells by staining for Annexin V [121], caspase activation [122] or by measuring the uptake of membrane impermeant fluorescent dyes such as propidium iodine (PI) or 7-7-amino-actinomycin D (7-AAD) which intercalate DNA when cell membrane integrity has been compromised [123]. Indirectly, flow cytometry can be used to measure CTL function by immunostaining T-cells for CD107a, a marker of T-cell degranulation [124,125] which suggests the release of perforin and granzyme B.

### 6.5. Systems Biology Approaches to Assessing Cellular Immunity

#### 6.5.1. DNA Microarray

The ability to measure complex host:pathogen interactions on an integrated global level using systems biology approaches provides powerful information about the temporal and spatial kinetics of infection as it progresses and resolves. These new technologies can be used to probe interactions at the DNA, small RNA (microRNA) or protein level. 

For routine microarray experiments, the requisite probes (e.g., oligonucleotide or DNA) are attached or directly synthesized onto the surface of a chip, typically glass, plastic or silicon but historically included various membrane types. Other commonly used microarray platforms use microscopic beads instead of the large solid chip support. The “target sample”, generally RNA isolated from requisite samples, is prepared such that downstream hybridization and subsequent detection of sample and probe binding is easily facilitated. During the incubation, complementary DNA strands will hybridize and this binding is typically detected using fluoro- or chemiluminescent methodologies. The strength of the signal, depends on the amount of target sample binding the probes present. Typical downstream bioinformatic analysis will investigate relative quantitation in which the intensity of target sample and probe binding is compared across various biological conditions. DNA microarrays can be used to measure changes in expression levels, to genotype, to detect single nucleotide polymorphisms (SNPs), to elucidate protein binding sequences, or to assay the expression of alternative splice forms, all of which can be used to inform many aspects of disease biology and vaccine development [126]. The use of transcriptomics to identify the cellular networks involved in cellular immune responses to influenza could be a highly sensitive tool in assessing correlates of protection in influenza vaccine trials. Such approaches have already proven useful in assessing the molecular factors which contribute to the development of humoral immune responses to influenza vaccines [127]. Importantly, a gene signature profile identified during a human influenza challenge study was used to develop a composite profile which could distinguish between naturally acquired swine-origin influenza A/H1N1 (2009) infected and non-infected individuals with 92% accuracy [128]. Currently, assessment of influenza vaccine efficacy in influenza challenge studies are dependent on descriptive and subjective classifications of clinical outcome, using symptom scores, often by volunteer self-reporting, which can be highly variable. A recent study by Davenport and colleagues, determined that by combining transcriptomic data with these clinical symptomatic scorings, variability in self-reporting of symptoms could be reduced [129]. The authors determined that in volunteers with severe laboratory confirmed influenza there was differential gene expression in approximately 1103 probes, which they subsequently reduced to six genes and successfully used differential expression of this limited cohort to predict symptomatic infection in an independent transcriptomics data set from a separate influenza challenge study [130]. One disadvantage of transcriptomics approaches is that mRNA levels do not always correlate with actual protein expression. Therefore, global expression can also be studied at the protein level by proteomics, and these approaches can be integrated with gene expression studies to provide more powerful investigations of host:pathogen interactions. 

#### 6.5.2. microRNA Profiling

MicroRNA are small non-coding RNAs which affect global gene expression networks and regulate the gene expression of hundreds of mRNAs through the active inhibition of mRNA translation or the promotion of mRNA destruction. [131]. As a result of their ability to regulate multiple mRNAs, the differential expression profiles of microRNAs are useful for capturing large amounts of informative bioinformatics. Advantageously, microRNAs can be measured with good sensitivity and there is the downstream potential for therapeutic alteration of cellular microRNA profiles in various disease states. Therefore, it is possible that vaccination approaches could be developed to preferentially stimulate microRNA profiles which correlate with protection or vaccine responsiveness.

There are numerous studies which have employed microarray or microRNA profiling in the study of influenza infection of lung epithelial cells [132]. Such studies have been shown to be capable of distinguishing between low-pathogenicity pandemic H1N1 and highly pathogenic avian-origin H7N7 [133]. Pre-clinical studies have shown that microRNAs in the lung may contribute to controlling acute influenza infection in pigs [134]. Importantly, recent studies have determined that approximately fourteen dysregulated microRNAs isolated from the whole blood of H1N1 influenza infected patients could clearly distinguish between infected and uninfected patients [135]. It will be advantageous to incorporate these powerful functional genomics tools into clinical assessments of novel influenza vaccine candidates in the future.

## 7. Summary of Differences in T-Cell Assays Used in Clinical Trials

In recent years the development of “universal” influenza vaccines which aim to stimulate cellular immunity have gained interest and a number of replication-deficient viral vectors (adenovirus and poxvirus) expressing relatively conserved influenza antigens have now been tested clinically [59,60,61,62,63,64], both in terms of safety and immunogenicity. In addition a Phase IIa study has been completed to test the efficacy of viral vectored influenza vaccines in human influenza challenge studies [62,64]. Other studies have measured the cellular response in controlled human influenza challenge studies [7,42] as well as characterising T-cell responses following natural influenza infection during pandemics [43]. Despite multiple studies which use T-cell assays to measure the role of CMI in influenza, there is no consensus on the phenotype or magnitude of the T-cell response as a correlate of protection. This is complicated by the fact that the field lacks standardized protocols for detecting cellular immunity and there are significant intra-laboratory differences in the way that T-cell assays are set up, with different types (e.g., whole protein, virion or peptide pools) or concentrations of antigen being used for stimulation of PBMCs, different numbers of cells being used per ELISPOT well, assays being performed with fresh or cryopreserved cells, locally established protocols and preferred reagents/suppliers. 

For example, in our influenza vaccine studies, ELISPOTs are performed on fresh PBMCs using 15–20 mer peptides overlapping by 10 which span the NP and M1 insert (derived from A/Panama/2007/99) within our viral vectored vaccines [59,60,61,62,63]. Cells (200,000) are stimulated for 18–20 h with 8 separate pools of 10 peptides at a final concentration of 10 µg/mL in a final volume of 100 µL. In three of our prior clinical studies, median IFN-γ ELISPOT responses at baseline in age groups 18–50 years were found to be 320 SFU/10^6^ PBMCs [60], 277 SFU/10^6^ PBMCs [63] and 188 SFU/10^6^ PBMCs in volunteers aged over 50 years [61]. Recently, Forrest and colleagues determined that a large proportion of infants and children with ≥100 SFU/10^6^ PBMCs, a value below what we estimated for baseline response in adults, were protected against clinical influenza and proposed that this ELISPOT value could be used as a measure of CMI in infants and children [45]. In the latter study, PBMCs were stimulated for 20–24 h with the inactivated monovalent vaccine components rather than peptides, although the number of cells and final concentration of stimulus is not provided. In a separate study, Wilkinson and authors reported that baseline T-cell responses to influenza as determined by ELISPOT were below 1000 SFU/10^6^ PBMCs when cells were stimulated with 18mer peptides overlapping by 10, spanning the full proteome of H1N1 (A/Brisbane/59/2004), H3N2 surface proteins (A/New York/388/2005) or H3N2 internal proteins (A/New York 232/2004), rather than just NP+M1 as in our vaccine studies [42]. With the exception of using 300,000 cells per well and peptides at a final concentration of 2 µg/mL, the authors used similar reagents, suppliers and protocols to our ELISPOT assay. Furthermore, using a fluorescence immunospot assay, Sridhar *et al.*, determined that the median baseline IFN^+^/IL-2^−^ response to live pH1N1 virus (152 SFU/10^6^ PBMCs) was higher than responses to conserved 9 mer peptides derived from PB1, NP or M1 (56 SFU/10^6^ PBMCs) [43]. Clearly, the baseline ELISPOT responses obtained when using peptide pools corresponding to individual influenza antigens, *versus* those that span the entire influenza proteome, or live influenza virus itself differ greatly and therefore these data cannot be compared directly to even indicate a baseline cellular immune response to influenza.

As baseline ELISPOT responses vary so greatly between published studies, it is currently difficult to predict what magnitude of IFN-γ production may be required to mediate protection following vaccination with a universal influenza vaccine, or indeed, how long the post-vaccination response should be maintained in order to protect. In a previous phase 1 study to assess the safety and immunogenicity of an MVA-NP+M1 influenza vaccine [63], peak median ELISPOT responses were 2793 SFU/10^6^ PBMCs and 2088 SFU/10^6^ PBMCs at day 7 and 21 post-vaccination following stimulation, in fresh PBMCs, representing a ~10-fold and 7.5-fold increase over baseline respectively (data not shown). Similarly, in volunteers aged 50+, one week following vaccination with the same MVA-NP+M1 vaccine, ELISPOT responses were increased ~8.5-fold to a median of 1603 SFU/10^6^ PBMCs. So that a potential correlate of protection may be defined, it may be more informative to report fold-changes between baseline and peak responses post-vaccination than to focus on specific ELISPOT values. Importantly, data obtained for immunogenicity in vaccination studies does not enable us to predict vaccine efficacy, and should be tested in the context of natural influenza infection or in influenza challenge studies conducted in human volunteers. Therefore, we carried out a Phase IIa influenza challenge study, where median baseline responses were 258 SFU/10^6^ PBMCs in the vaccine group and 300 SFU/10^6^ PBMCs in the controls [62]. Following vaccination, these responses were elevated to 980 SFU/10^6^ PBMCs by day 21 post-vaccination (~3.7-fold increase) but declined to 627 SFU/10^6^ PBMCs by the day prior to influenza challenge, which was ~2.4-fold above baseline. Although fewer vaccinated volunteers developed laboratory confirmed symptoms of influenza, a defined correlate of protection by IFN-γ ELISPOT was not determined from this small study [62]. 

Similarly, many discrepancies exist when reporting ICS results making it difficult to determine if it is the T-cell phenotype, functionality or magnitude of the response that may provide correlates of protection for influenza. In a human challenge study, Wilkinson and colleagues identified that pre-existing CD4^+^ responding to internal, relatively highly conserved proteins were associated with reduced viral shedding and disease severity in H3N2/H1N1 seronegative volunteers [42]. H3N2 and H1N1 were the influenza challenge strains used in the study. Cellular responses, as measured by IFN-γ were largely CD4^+^ (56%) and responses to H3N2 nucleoprotein and matrix peptides were immunodominant, with 57% (8/14) and 50% (7/14) of subjects responding respectively. For H1N1, responses to matrix were immunodominant (6/9; 78%) and again, largely CD4^+^ (72%). On day 7 post-challenge, the breadth (number of influenza proteins recognized) and proportion of the T-cell response to influenza peptides was found to be increased approximately 10-fold. Importantly however, immunodominant T-cell responses to NP and M1 persisted to D28 post-challenge whereas others were diminished once the acute phase had finished. 

In contrast, in a natural setting of influenza infection within the community, Sridhar and colleagues measured the responses of T-cells to influenza prior to, and during, the 2nd/3rd waves of the pH1N1 pandemic [43]. The authors determined that it was pre-existing, functional CD8^+^IFN-γ^+^IL-2^−^/CD45RA^+^/CCR7^−^ T-cells that were inversely correlated with symptom severity in individuals who became infected with influenza virus. Furthermore, they determined that the CD8^+^IFN-γ^+^ population had lung homing potential (CCR5^+^) and cytotoxic capacity (CD107a/b) in response to live influenza virus, as assessed through multiparameter flow cytometry. In this study, PBMCs were stimulated with whole influenza virus [MOI 5 (A/England/09/195)] for 18 h so as to maintain consistency with the timing of the ELISPOT assay and monesin A was added 2 h after addition of stimulus [43]. 

In our vaccination studies with MVA-NP+M1, we determined that the phenotype of T cells responding to NP+M1 peptide pools were largely CD8^+^, with more antigen-specific CD3^+^CD8^+^ T cells than CD3^+^CD4^+^ T cells producing IL-2, TNF-α, and CD107a following vaccination [63]. Importantly, these responses were polyfunctional, as determined by CD107a staining and the production of IFN-γ, TNF-α and IL-2 [61]. More detailed T-cell clonality studies using the GILGFVFTL/HLA A*0201 M1-restricted tetramer indicated that MVA-NP+M1 expanded pre-existing memory CD8^+^ T-cells, which displayed a predominant CD45RO^+^CD27^+^CD57^−^CCR7^−^ M1-specific phenotype both before and after vaccination in older adults [61].

The limited number of influenza challenge studies and community-wide studies during influenza pandemics make it difficult to conclusively identify a specific T-cell phenotype which correlates with protection. This complicates efforts to develop vaccines which aim to stimulate CMI. Furthermore, it is important to cautiously interpret the protective role that peripheral T-cell responses may play during human influenza infection, in the absence of analysis of mucosal resident T-cell responses. It is clear that more detailed phenotyping of lung homing or lung resident T-cells, which can now be performed [136], as well as investigation of the role of T-cell mediated protection in the presence of influenza-specific neutralizing antibody responses, could also be used to improve rational vaccine design.

## 8. Conclusions

It is evident that standardization of all aspects of cellular assays will be required in order to advance our understanding of the T-cell mediated correlates of protection in influenza infection. This information would be highly informative when screening various “universal influenza vaccine” candidates. Efforts to standardize protocols for the cryopreservation of PBMCs, storage and validation of end-point assays across multiple sites have already been conducted in large scale clinical trials for HIV [137,138,139]. These efforts have included the establishment of repositories or cell banks for PBMCs which can be used in multiple sites and in replicate assays to maintain consistency [139]. Importantly, it has been demonstrated that the ELISPOT assay can be standardized for the measurement of cellular immunity in HIV clinical trials in multiple laboratories based on different continents [140]. 

Similar consortia, such as the Consortium for the Standardization of Influenza Seroepidemiology (CONSISE http://consise.tghn.org) have been established within the influenza field to standardize serological assays including the haemagglutination inhibition assay and the microneutralization assay. However, no coordinated effort has established standardized SOPs for assays of cellular responses to influenza. The ELISpot assay can be adapted to study the response to individual epitopes, whole virus antigens or whole viruses, making it suitable for use in clinical trials of different candidate vaccines. Vaccine efficacy trials (phase III) for influenza vaccines are large and expensive studies. Therefore, it is important to establish protocols which may be implemented in clinical vaccine immunogenicity trials (phase II), allowing comparison of the cell-mediated response to different vaccines at an earlier stage. This will allow prioritization of vaccine candidates for efficacy studies. This concerted effort to standardize cellular assays for influenza will facilitate marked progress in the development of a universal influenza vaccine.
